# Inhaled nitric oxide and acute kidney injury risk: a meta-analysis of randomized controlled trials

**DOI:** 10.1080/0886022X.2021.1873805

**Published:** 2021-01-25

**Authors:** Junqiu Wang, Xuhui Cong, Mengrong Miao, Yitian Yang, Jiaqiang Zhang

**Affiliations:** a Journal Editorial Department, Henan Provincial People’s Hospital, People’s Hospital of Zhengzhou University, Zhengzhou, China; b Department of Anesthesiology and Perioperative Medicine, Henan Provincial People’s Hospital, People’s Hospital of Zhengzhou University, Zhengzhou, China

**Keywords:** Inhaled nitric oxide, acute kidney injury, randomized controlled trial, acute respiratory distress syndrome, cardiac surgery, organ transplantation

## Abstract

**Purpose:**

There are conflicting results as to the effect of inhaled nitric oxide (iNO) therapy on the risk of acute kidney injury (AKI). The aim of this study was to perform a meta-analysis to assess the updated data.

**Methods:**

We systematically searched Web of Science, the Cochrane Library, Wanfang, and PubMed for relevant randomized control trials between database inception and 9/07/2020. Relative risks (RRs) with 95% confidence intervals (CIs) predicting the risk of AKI were extracted to obtain summary estimates using fixed-effects models. The Trim and Fill method was used to evaluate the sensitivity of the results and adjust for publication bias in meta-analysis.

**Results:**

15 randomized controlled studies from 14 articles involving 1853 patients were included in the study. Analyzing the eligible studies we found: (1) iNO therapy significantly increased the risk of AKI in acute respiratory distress syndrome patients (RR 1.55, 95% CI 1.15–2.10, *p* = 0.004; *I*^2^ for heterogeneity 0%; *P*_het_ = 0.649). (2) The use of iNO was associated with reduced AKI risk in patients undergoing cardiac surgery (RR 0.80, 95% CI 0.64–0.99, *p* = 0.037; *I*^2^ for heterogeneity 0%; *P*_het_ = 0.528). (3) For organ transplantation recipients, there was no effect of iNO administration on the risk of AKI (RR 0.50, 95% CI 0.16–1.56, *p* = 0.233; *I*^2^ for heterogeneity 0%; *P*_het_ = 0.842). The Trim and Fill analysis showed that the overall effect of this meta-analysis was stable.

**Conclusions:**

The effect of iNO on AKI risk might be disease-specific. Future RCTs with larger patient populations should aim to validate our findings.

## Introduction

Nitric oxide (NO) is an important signaling substance and vasodilator. It can relax vascular smooth muscle cells as well as pericytes by binding to the heme moiety of cytosolic guanylate cyclase and ultimately causing a fall in intracellular Ca^2+^ [1]. Inhalation of NO (iNO) leads to selective pulmonary vasodilatation and reduces pulmonary vascular resistance, increases arterial oxygenation, and improves pulmonary angiogenesis and lung alveolarization [2]. Given these beneficial effects, iNO is used widely as therapy in the field of critical care and medicine in general, including acute respiratory distress syndrome (ARDS), neonatal pulmonary hypertension, and cardiac surgery.

Although iNO has excellent effectiveness and safety profile, its potential adverse effects should not be neglected. Two previous meta-analyses raised concerns about the relationship between the use of iNO and the risk for acute kidney injury (AKI) [3,4]. In 2007, Adhikari and colleagues included four randomized control trials (RCTs) involving 895 participants in a pooled analysis and suggested that iNO increased the risk for renal dysfunction (risk ratio 1.50, 95% confidence interval 1.11–2.02) [3]. Another meta-analysis published in 2015 by Ruan et al. [4] found that the use of iNO was associated with higher renal dysfunction risk especially with prolonged use in ARDS patients. Despite these interesting findings, there were some limitations to the meta-analyses. First, a sensitivity analysis was not performed and the stability of the results were not evaluated. Second, most included studies in these meta-analyses focused on ARDS and there was less evidence on other diseases. Third, the number of relevant researches has increased rapidly since these meta-analyses were published. The accumulating evidence needs to be reevaluated. Therefore, we aimed to perform an up-dated quantitative assessment of the relationship between iNO and AKI.

## Methods

This meta-analysis was conducted and reported according to the Preferred Reporting Items for Systematic Reviews and Meta-Analyses statement [5]. There was no registered protocol for this meta-analysis.

### Search methods

Applying a predetermined search strategy, two independent investigators (JW and XC) searched Web of Science, the Cochrane Library, Wanfang, and PubMed in order to identify potentially relevant articles between database inception and 9 July 2020. The following search terms were used: ‘inhaled nitric oxide’ and ‘randomized controlled trial’. Secondary searching included a manual search of reference lists in previous meta-analyses, reviews, and all included studies. There were no language restrictions. The search details are shown in Supplementary Table S1.

### Inclusion and exclusion criteria

Studies were included in this meta-analysis if they met the following inclusion criteria: (1) the eligible studies were RCTs; (2) they compared iNO with placebo or usual treatment; and (3) the number of patients with renal dysfunction was reported in iNO and control groups. The exclusion criteria were: (1) retrospective studies, cohort studies, and non-randomized controlled studies; (2) conference abstracts; and (3) data for renal dysfunction were not reported.

### Data extraction

From all RCTs found eligible, the following information was extracted: the family name of the first author, country/region of research, year of publication, study designs and methods, participant number and details, intervention details, and the number of patients with renal dysfunction in iNO and control groups. Two investigators (JW and XC) performed data extraction. Any disagreements were resolved by discussion between the two investigators and JZ where necessary.

### Assessment for risk of bias

Two investigators (JW and XC) assessed the risk of bias for each trial by using criteria according to the Cochrane Risk of Bias Tool, which allows for evaluating six domains: method of random sequencing, allocation concealment, blinding of participants and personnel, blinding of outcome assessors, incomplete outcome data, selective reporting, and other factors that may affect bias [6]. Discrepancies were resolved by discussion or by consultation with the third investigator, JZ.

### Meta-analyses

For dichotomous data, we used risk ratios (RRs) as the effect measure with 95% confidence intervals (CIs) calculated using the fixed-effects model, where a RR > 1 indicates an increased likelihood of renal dysfunction when treated with iNO compared with placebo. We generated summary forest plots to show the RRs and 95% CIs. Statistical heterogeneity between trial results was assessed using the *I*^2^ statistic and the Chi-square test. It was classified as large (75%), moderate (50%), and low (25%) [7]. We considered it to be substantial heterogeneity when the *I*^2^ was greater than 50% and was accompanied by a statistically significant Chi^2^ statistic. The cause of heterogeneity was explored using subgroup analyses. Finally, publication bias was evaluated by graphical analysis of the funnel plot. Egger’s regression test was used to test the funnel plot [8]. All analyses were performed in Stata software, version 12.0 (College Station, TX). The significance level for all statistical tests was *p* < 0.05 (two-tailed).

## Results

**Figure 1.**
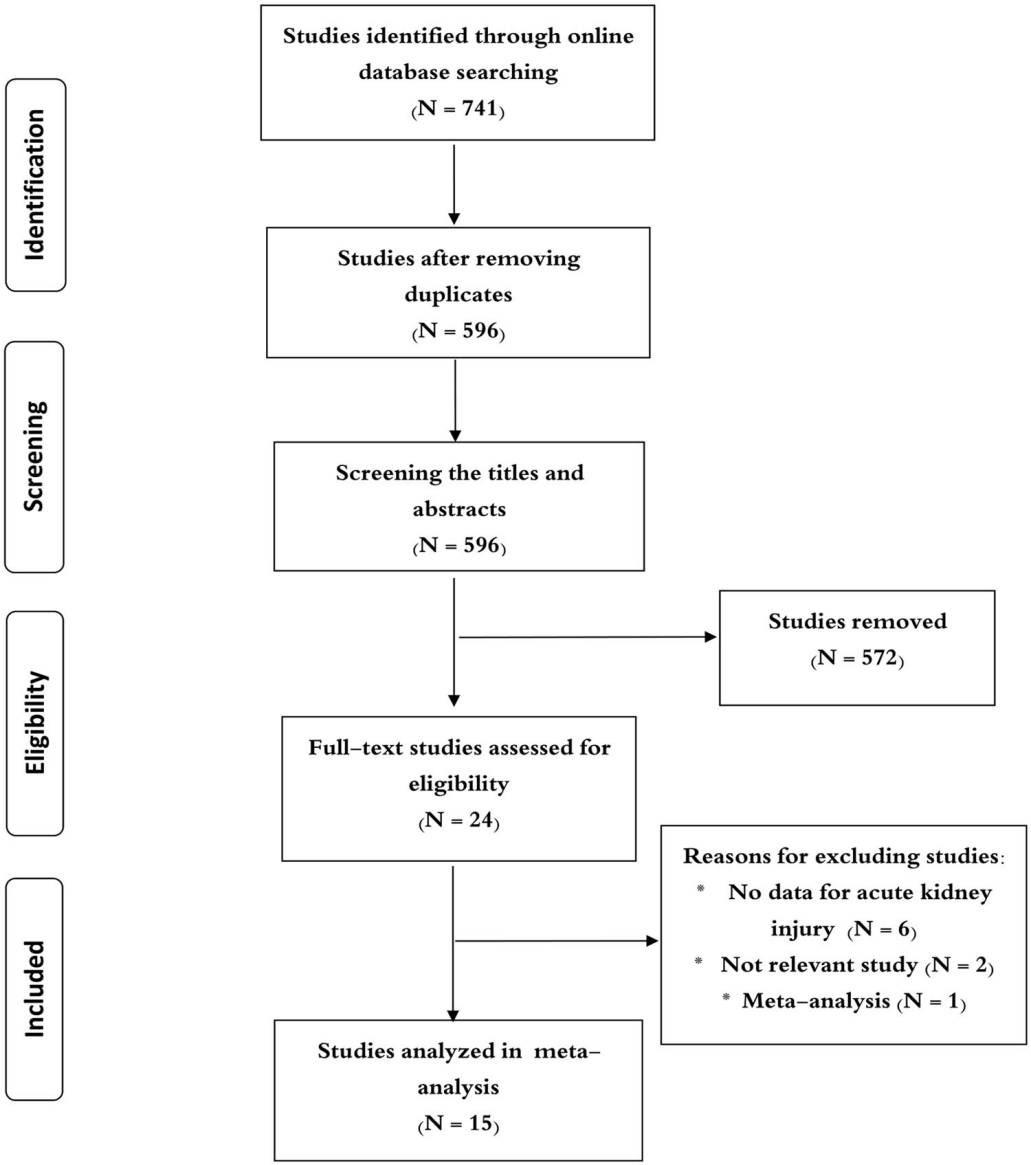
Flow chart for identification of studies in the meta-analysis.

The literature search yielded 741 articles. After the removal of duplicates, they were reduced to 596. At the screening of study titles and abstracts, an additional 572 articles were excluded as they did not meet our inclusion criteria. Figure 1 shows how we picked the studies. Ultimately, a total of 15 studies from 14 articles were analyzed in our final meta-analysis [9–22]. All studies were performed in North America, Europe, China, Brazil, and Uganda. Among the included studies, 4 studies reported the results for ARDS [9,11–13], 4 studies reporting cardiac surgery [15,16,21,22], 3 studies reporting organ transplantation [14,17], and 4 studies reporting other diseases [10,18–20]. The number of participants in each trial varied with 29 subjects in the smallest study and 385 subjects in the largest study. The majority were men, except in one study. All the studies provided data for AKI. Table 1 shows these studies selected for the final meta-analysis. Detailed risk of bias assessments are presented in Table 2.

**Table 1.** Characteristics of included studies.

Trial	Location	*n*	Men (%)	Age	Intervention description	AKI definition	Number of AKI patients		RR with 95% CI	*p*-Value	Disease
							iNO	Control			
Dellinger et al. 1998 [9]	USA	iNO: 120 Control: 57	iNO: 64 Control: 70	iNO: 49 ± 18 years Control: 47 ± 18 years	iNO at concentrations of 1.25, 5, 20, 40, or 80 ppm for up to 28 days or till FiO_2_ < 0.5	Creatinine above 2 mg/dL	20	7	1.36 (0.61–3.02)	0.448	ARDS
Lundin et al. 1999 [11]	11 European countries	iNO: 80 Control: 74	iNO: 75 Control: 62	iNO: 58 ± 15 years Control: 56 ± 17 years	1–40 ppm of NO at the lowest effective dose for up to 30 days or until an end point was reached	Creatinine above 300 mmol/L and/or institution of RRT	28	12	2.16 (1.19–3.92)	0.008	ARDS
Kinsella et al. 1999 [10]	USA	iNO: 48 Control: 32	iNO: 58 Control: 63	iNO: Gestational 27.1 weeks Control: Gestational 26.8 weeks	5 ppm of NO for up to 7 days	Acute renal failure	2	2	0.67 (0.10–4.49)	0.675	Severe hypoxaemic respiratory failure
Payen et al. 1999 [12]	European countries	iNO: 98 Control: 105	Unknown	Adults	10 ppm of NO for 5 days	Requiring RRT	33	26	1.36 (0.88–2.10)	0.162	ARDS
Taylor et al. 2004 [13]	USA	iNO: 192 Control: 193	iNO: 52 Control: 54	iNO: 50 ± 17 years Control: 50 ± 17 years	5 ppm of NO until 28 days	Creatinine above 265.2 µmol/L	12	8	1.51 (0.63–3.61)	0.352	ARDS
Perrin et al. 2006 [14]	France	iNO: 15 Control: 15	iNO: 60 Control: 53	iNO: 37 ± 12 years Control: 33 ± 11 years	20 ppm of NO during a 12-h period	Requiring RRT	1	1	1.00 (0.07–14.55)	1.000	Lung transplantation
Fernandes et al. 2011 [15]	Brazil	iNO: 14 Control: 15	iNO: 7 Control: 20	iNO: 48 ± 11 years Control: 44 ± 13 years	10 ppm of NO for 48 h	Acute renal failure	0	1	0.36 (0.02–8.07)	0.517	Cardiac surgery
Potapov et al. 2011 [16]	Germany and USA	iNO: 73 Control: 77	iNO: 88 Control: 84	iNO: 58 ± 10 years Control: 54 ± 12 years	40 ppm of NO for 48 h	Requiring RRT	10	8	1.32 (0.55–3.16)	0.533	Cardiac surgery
Lang et al. 2014 (A) [17]	USA	iNO: 20 Control: 20	iNO: 80 Control: 80	iNO: 54 (36–72) years Control: 55 (45–68) years	80 ppm of NO during the operative phase of liver transplantation	Renal dysfunction	2	5	0.40 (0.09–1.83)	0.212	Liver transplantation
Lang et al. 2014 (B) [17]	USA	iNO: 20 Control: 20	iNO: 75 Control: 85	iNO: 59 (30–67) years Control: 57 (24–70) years	80 ppm of NO during the operative phase of liver transplantation	Renal dysfunction	1	2	0.50 (0.05–5.08)	0.548	Liver transplantation
Trzeciak et al. 2014 [18]	USA	iNO: 26 Control: 23	iNO: 42 Control: 57	iNO: 59 ± 15 years Control: 58 ± 20 years	40 ppm of NO for 6 h	Requiring RRT	2	1	1.77 (0.17–18.26)	0.626	Severe sepsis or septic shock
Hawkes et al. 2015 [19]	Uganda	iNO: 88 Control: 92	iNO: 60 Control: 53	iNO: 2 (1–3) years Control: 2 (1–3) years	80 ppm of NO for up to 72 h	Serum creatinine >1.5 × ULN (children age 1–2) or >2.0 × ULN (age 2–10) AND an abrupt (within 48 h) reduction in kidney function	7	3	2.44 (0.65–9.14)	0.169	Severe malaria
Liu et al. 2016 [20]	China	iNO: 19 Control: 13	iNO: 68 Control: 62	iNO: Gestational 37.4 weeks Control: Gestational 36.1 weeks	15–20 ppm of NO for 96 h	Acute renal failure	1	0	2.10 (0.09–47.89)	0.642	Hypoxic respiratory failure
Lei et al. 2018 [21]	China	iNO: 117 Control: 127	iNO: 44 Control: 41	iNO: 49 ± 10 years Control: 48 ± 9 years	80 ppm of NO for 24 h or less if patients were ready to be extubated early.	An increase in serum creatinine by 50% within 7 days of surgery, or an increase in serum creatinine by 0.3 mg/dL within 2 days of surgery from preoperative baseline levels of serum creatinine	58	81	0.78 (0.62–0.97)	0.025	Cardiac surgery
Kamenshchikov et al. 2019 [22]	Russia	iNO: 30 Control: 30	iNO: 83 Control: 70	iNO: 62 years Control: 58 years	40 ppm of NO during CPB	Unknown	1	3	0.33 (0.04–3.03)	0.301	Cardiac surgery

AKI: acute kidney injury; ARDS: acute respiratory distress syndrome; CPB: cardiopulmonary bypass; NO: nitric oxide; RRT: renal replacement therapy; ULN: upper limit of normal.

**Table 2.** Assessment of risk of bias in individual studies.

Trial	Sequence generation	Allocation concealment	Blinding	Incomplete outcome data	Selective outcome reporting	Other sources of bias
Dellinger et al. [9]	Low risk	Low risk	Low risk	Low risk	Low risk	Low risk
Lundin et al. [11]	Low risk	Low risk	High risk	Low risk	Low risk	High risk
Kinsella et al. [10]	Low risk	Low risk	Low risk	Low risk	Low risk	Low risk
Payen et al. [12]	Low risk	Low risk	Low risk	Low risk	Low risk	High risk
Taylor et al. [13]	Low risk	Low risk	Low risk	Low risk	Low risk	Unclear risk
Perrin et al. [14]	High risk	Unclear risk	High risk	Low risk	Low risk	Unclear risk
Fernandes et al. [15]	Low risk	Low risk	Low risk	Low risk	Low risk	Low risk
Potapov et al. [16]	Low risk	Low risk	Low risk	Low risk	Low risk	Low risk
Lang et al. (A) [17]	Low risk	Low risk	Low risk	Low risk	Low risk	Low risk
Lang et al. (B) [17]	Low risk	Low risk	Low risk	Low risk	Low risk	Low risk
Trzeciak et al. [18]	Low risk	Low risk	Low risk	Low risk	Low risk	Low risk
Hawkes et al. [19]	Low risk	Low risk	Low risk	Low risk	Low risk	Low risk
Liu et al. [20]	High risk	Unclear risk	Low risk	Low risk	Low risk	Low risk
Lei et al. [21]	High risk	Low risk	Low risk	Low risk	Low risk	Unclear risk
Kamenshchikov et al. [22]	Low risk	Low risk	Low risk	Low risk	Low risk	Low risk

**Figure 2.**
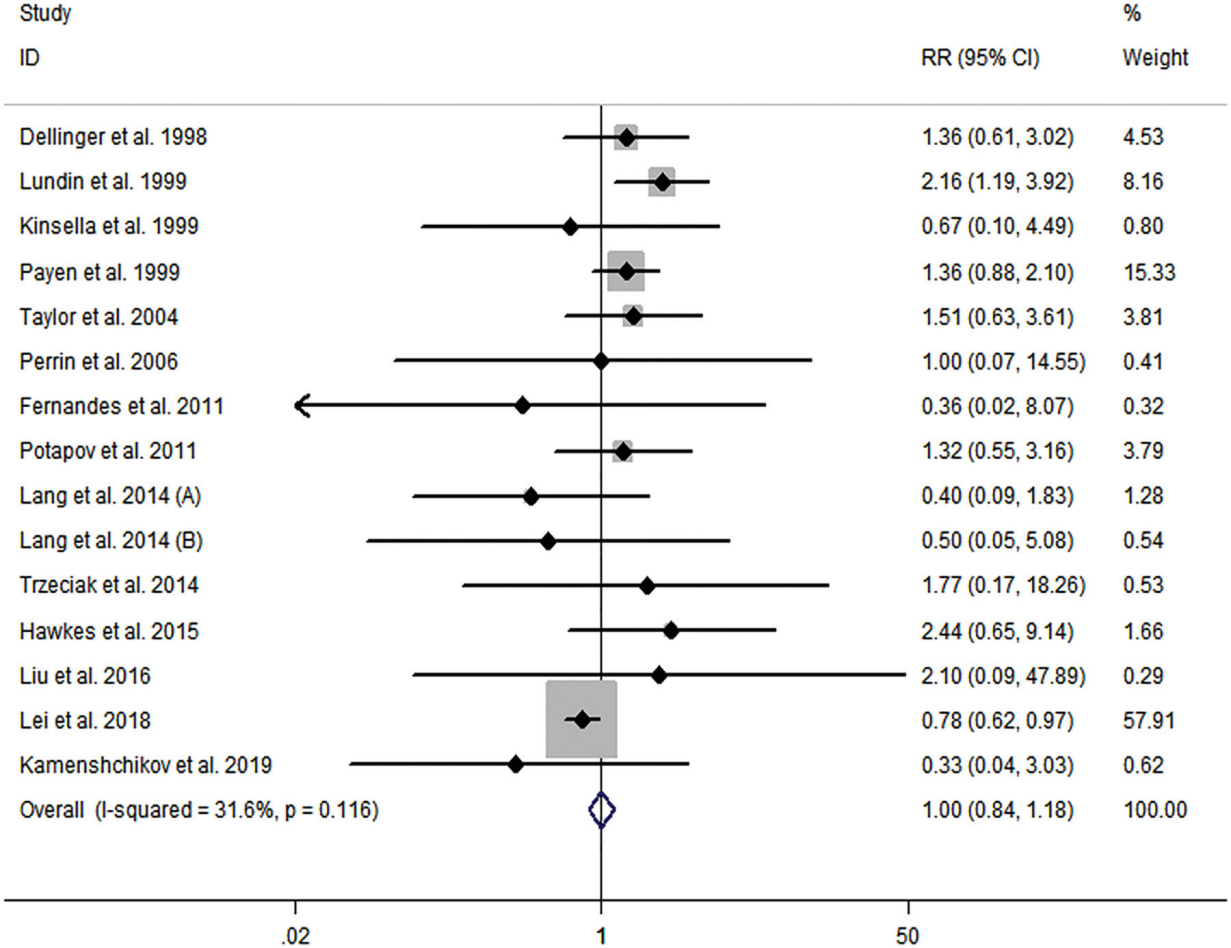
Meta-analysis of the effect of inhaled nitric oxide on acute kidney injury risk by pooling the 15 randomized controlled trials.

**Figure 3.**
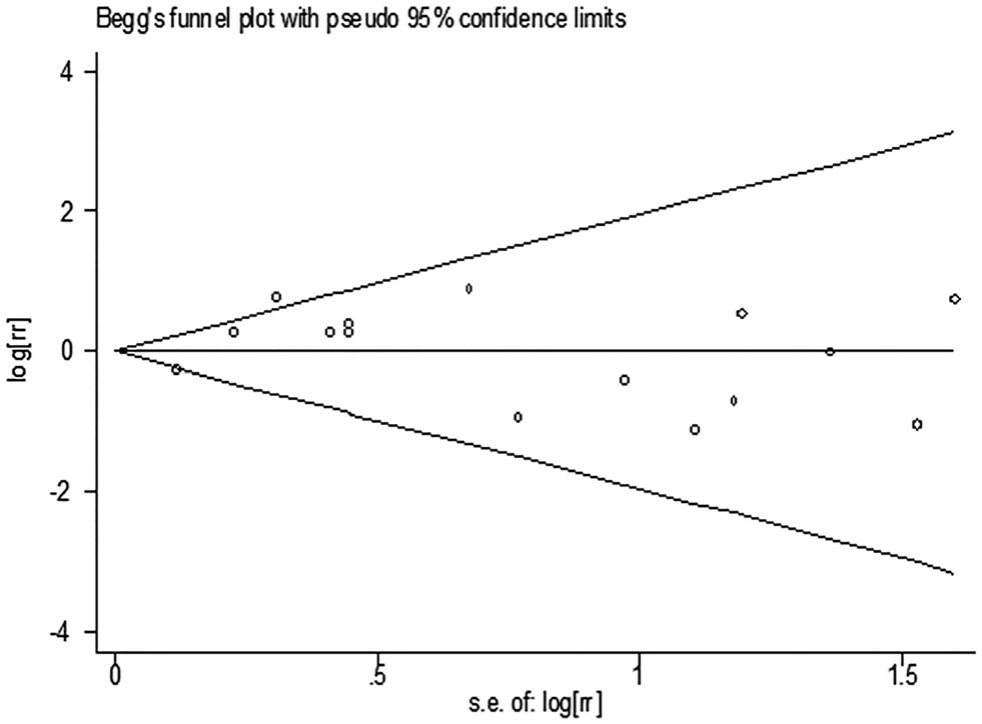
Begg’s funnel plot for evaluating publication bias.

**Figure 4.**
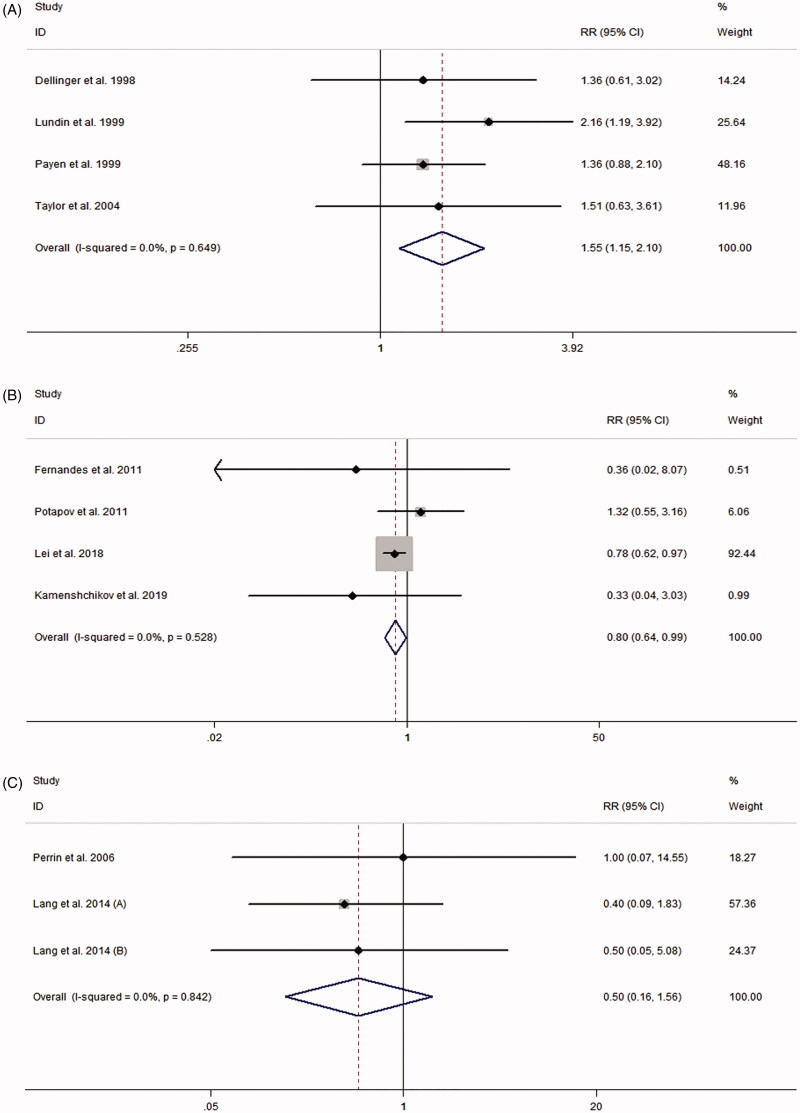
Meta-analysis of the effect of inhaled nitric oxide on acute kidney injury risk. (A) Acute respiratory distress syndrome. (B) Cardiac surgery. (C) Organ transplantation.

Table 1 presents the RRs for AKI from each study. The pooled meta-analysis showed that iNO treatment was not associated with AKI risk, with a pooled RR of 1.00 (95% CI 0.84–1.18; *p* = 0.977; *I*^2^ for heterogeneity 31.6%; *P*_het_ = 0.116) (Table 3 and Figure 2). Egger’s test (*p* = 0.461), Begg’s test (*p* = 0.198), and visual evaluation of the funnel plot (Figure 3) indicated no publication bias. In subgroup analysis based on the fixed-effects model, the use of iNO was associated with increased risk of AKI in ARDS patients (RR 1.55, 95% CI 1.15–2.10, *p* = 0.004; *I*^2^ for heterogeneity 0%; *P*_het_ = 0.649) (Table 3 and Figure 4(A)), but iNO treatment decreased AKI risk for individuals who received cardiac surgery (RR 0.80, 95% CI 0.64–0.99, *p* = 0.037; *I*^2^ for heterogeneity 0%; *P*_het_ = 0.528) (Table 3 and Figure 4(B)). In addition, there was no effect of iNO therapy on the risk of AKI in patients undergoing organ transplantation (Table 3 and Figure 4(C)). The robustness of the results was evaluated by a sensitivity analysis using the trim and fill method [23]. As shown in Table 4, the results of the trim and fill test showed that sensitivity analyses for ARDS and cardiac surgery remained significant.

**Table 3.** Meta-analysis evaluation of the relationship between inhaled nitric oxide and acute kidney injury risk.

Subgroup	Number of studies	RR (95% CI)	*p*	Heterogeneity	
				*I*^2^	*P*_het_
All	15	1.00 (0.84–1.18)	0.977	31.6%	0.116
ARDS	4	1.55 (1.15–2.10)	0.004	0	0.649
Cardiac surgery	4	0.80 (0.64–0.99)	0.037	0	0.528
Organ transplantation	3	0.50 (0.16–1.56)	0.233	0	0.842
Others	4	1.67 (0.65–4.27)	0.286	0	0.747

ARDS: acute respiratory distress syndrome; CI: confidence interval; RR: relative risk.

**Table 4.** Trim and Fill test results.

Subgroup	Model	RR (95% CI)	*p*	Heterogeneity	
				*I*^2^	*P*_het_
ARDS	Fixed-effect	1.55 (1.15–2.10)	0.005	0	0.651
	Random-effect	1.55 (1.15–2.10)	0.005	0	0.651
Cardiac surgery	Fixed-effect	0.80 (0.64–0.99)	0.037	0	0.528
	Random-effect	0.80 (0.64–0.99)	0.037	0	0.528
Organ transplantation	Fixed-effect	0.40 (0.15–1.05)	0.061	0	0.914
	Random-effect	0.40 (0.15–1.05)	0.061	0	0.914

ARDS: acute respiratory distress syndrome; CI: confidence interval; RR: relative risk.

### Heterogeneity

To quantify the between-study heterogeneity, we utilized the *I*^2^ statistic and the Chi-square test. There was no significant heterogeneity across the studies in both the overall meta-analysis and subgroup analyses. This enabled us to pool evidence from the included studies using the fixed-effects model.

## Discussion

We carried out a meta-analysis by combining evidence from 15 randomized clinical studies to evaluate the effect of iNO therapy on the risk of AKI. The main findings of the meta-analysis are the following: (i) iNO therapy significantly increased the risk of AKI in ARDS patients; (ii) the use of iNO was associated with reduced AKI risk in patients undergoing cardiac surgery; (iii) for organ transplantation recipients, there was no effect of iNO administration on the risk of AKI.

It is widely accepted that the effects of NO are limited to the lungs when delivered by inhalation. Many published RCTs suggest that iNO is associated with a very low incidence of adverse effects in the usual range of dosage. However, because iNO forms many metabolites that can act as endocrine carriers of NO in circulation, the safety aspects of iNO remain the focus of research. Lundin and coworkers focused on the adverse effects of iNO on ARDS in a European multicentre study [11]. They found that although iNO administration was not associated with bleeding complications, marked methemoglobinemia, or increased frequency of pneumothorax, there was an association between acute renal failure and iNO in ARDS (RR 2.16, 95% CI 1.19–3.92) [11]. This raised a concern about iNO-related renal dysfunction in ARDS patients. Several other studies from the USA and Europe also evaluated this possible relationship, including Dellinger et al. [9], Payen et al. [12], and Taylor et al. [13]. Based on the findings, two meta-analyses reviewed the data and concluded that the use of iNO increased AKI risk on ARDS [3,4]. Our meta-analysis confirmed the association between iNO administration and renal dysfunction in ARDS patients (RR 1.55). In addition, using the trim and fill method (Table 4), the meta-analysis results were shown to be stable, and no evidence of publication bias was detected. Previous meta-analyses and ours highlighted the potential AKI risk associated with iNO therapy in ARDS. It remains unknown why iNO administration in ARDS is linked to increased AKI risk. However, it is commonly believed that prolonged treatment with iNO might cause detrimental cell damage by reacting with reactive oxygen species and the resulting formation of reactive nitrogen species [24]. NO oxidative products can oxidize DNA bases and create DNA strand damage [25]. They also disrupt the maintenance of oxidation-sensitive enzymes, inhibit mitochondrial respiration, induce nuclear factor-kappaB-mediated protein degradation, and enhance caspase activation [26,27]. Dellinger and coworkers found a positive link between iNO doses and circulating levels of NO oxidative products (such as NO_2_) in patients with ARDS [9]. There is evidence that the generation of NO oxidative products contributed to glomerular cell apoptosis [28]. In addition, animal studies showed that the prolonged administration of iNO could induce apoptosis of the cells of the collecting ducts and the distal convoluted tubular cells, leading to renal injury [29]. In a rat model of diabetic kidney disease, endothelial nitric oxide synthase (eNOS) and inducible nitric oxide synthase (iNOS)-derived high NO production and oxidative stress contributed to apoptosis in the kidney [30]. However, the precise mechanisms of iNO-induced AKI in ARDS patients remain largely unknown, deserving further research in the future.

Cardiac surgery is a procedure that is commonly carried out in the world. Despite promising technical advances, it is still a high-risk surgery. AKI is a common and serious complication post cardiac surgery. AKI has been found in approximately 40–70% of patients undergoing cardiac surgery and is associated with an unfavorable prognosis [31]. The primary mechanisms of cardiac surgery-associated AKI include hemolysis, renal ischemia, and inflammation [31]. Unlike patients with ARDS, clinical studies showed that patients undergoing cardiac surgery developed a NO deficient state due to hemolysis. NO depletion induces a proinflammatory cascade, causes oxidative stress, produces vasoconstriction, and impairs endothelial function and tissue perfusion [32]. Preclinical and clinical data demonstrated that NO was a renal-protective agent during hemolysis and might thus prevent cardiac surgery-associated AKI. In a canine model of water-induced hemolysis, the use of iNO significantly reversed the vasoconstrictor effect of hemolysis, reduced serum creatinine, and attenuated renal impairment [33]. Several RCTs evaluated the effect of iNO therapy on renal dysfunction in patients undergoing cardiac surgery. In a Chinese RCT of 244 patients undergoing multiple valve replacement, Lei and coworkers found that NO administration during cardiopulmonary bypass and for the first 24 h postoperatively was associated with a decreased incidence of postoperative AKI and major adverse kidney events [21]. The trial by Lei et al. also indicated that the nephroprotective effect of iNO might be attributed to iNO’s impact on hemodynamics and right ventricular afterload. Their findings were supported by a recent European study in which Lomivorotov et al. demonstrated cardioprotective and nephroprotective effects of NO administration in cardiac surgery [22]. Combining results from different RCTs, this meta-analysis indicated a 20% decreased risk of postoperative AKI in cardiac surgery patients who received iNO therapy. Our results supported a previous meta-analysis by Hu et al. [34]. It is worth mentioning that several RCTs (such as NCT02836899 and NCT03527381) are underway to further assess the nephroprotective effect of NO in cardiac surgery. These trials may help to elucidate the underlying mechanisms of NO therapy for protecting AKI post-cardiac surgery.

In addition to ARDS and cardiac surgery, our meta-analysis also evaluated the effect of iNO on AKI incidence in other conditions, including organ transplantation. Some animal studies and RCTs reported that administrating iNO during the intraoperative period had protective effects on ischemia-reperfusion induced injury post-transplantation [14,17,35,36]. However, the results of our meta-analysis did not show any difference in the incidence of AKI post-transplantation between the iNO group and the control group.

Our meta-analysis has several limitations. Firstly, it remains unclear if the effect of iNO therapy on AKI risk in different diseases is dose-related. Although there was no significant heterogeneity in our meta-analysis, the included studies did vary in study design and iNO administration, making it difficult to assess dose-related effects. Secondly, most of the included studies were performed in adults and the number of pediatric studies was small. There were only three pediatric studies on the topic. It requires more research to investigate the effect of iNO therapy on renal function and AKI in pediatric patients. Thirdly, the incidence of AKI was not constantly reported by RCTs evaluating the effect of iNO therapy. Although Egger’s test did not suggest the presence of publication bias, we further performed the Trim and Fill test to assess the sensitivity of the results and adjust for publication bias in meta-analysis. As shown in Table 4, the results indicated that the overall effect of this meta-analysis was stable.

In conclusion, this meta-analysis suggested that iNO therapy increased the risk of AKI in ARDS patients, but the use of iNO was associated with a decreased risk of AKI in patients undergoing cardiac surgery. For organ transplantation recipients, iNO therapy had no effects on AKI incidence post-transplantation. Future RCTs are necessary to evaluate if the effects of iNO on AKI incidence is disease-specific. In addition, future RCTs should focus on the relation of iNO duration and dose with renal function.

## Supplementary Material

Supplemental Material
