# Supplementary material for: Inhaled nitric oxide and acute kidney injury risk: a meta-analysis of randomized controlled trials
Source: Ren Fail. 2021 Jan 25;43(1):281–90. doi: 10.1080/0886022X.2021.1873805 (PMC7850389; doi:10.1080/0886022X.2021.1873805)
Supplement: Supplemental Material [file IRNF_A_1873805_SM2179.pdf]

Supplementary table S1 Search in each database.

Database	Keywords
PubMed	"inhaled nitric oxide" and "randomized controlled trial" ("administration, inhalation"[MeSH Terms] OR ("administration"[All Fields] AND "inhalation"[All Fields]) OR "inhalation administration"[All Fields] OR "inhalant"[All Fields] OR "inhalability"[All Fields] OR "inhalable"[All Fields] OR "inhalants"[All Fields] OR "inhalated"[All Fields] OR "inhalation"[MeSH Terms] OR "inhalation"[All Fields] OR "inhal"[All Fields] OR "inhalations"[All Fields] OR "inhale"[All Fields] OR "inhaled"[All Fields] OR "inhaling"[All Fields] OR "inhalational"[All Fields] OR "inhalative"[All Fields] OR "inhalatively"[All Fields] OR "inhalent"[All Fields] OR "inhaler s"[All Fields] OR "inhales"[All Fields] OR "nebulizers and vaporizers"[MeSH Terms] OR ("nebulizers"[All Fields] AND "vaporizers"[All Fields]) OR "nebulizers and vaporizers"[All Fields] OR "inhalator"[All Fields] OR "inhalators"[All Fields] OR "inhaler"[All Fields] OR "inhalers"[All Fields]) AND ("nitric oxide"[MeSH Terms] OR ("nitric"[All Fields] AND "oxide"[All Fields]) OR "nitric oxide"[All Fields]) AND ("randomized controlled trial"[Publication Type] OR "randomized controlled trials as topic"[MeSH Terms] OR "randomized controlled trial"[All Fields] OR "randomised controlled trial"[All Fields])
Web of Science	"inhaled nitric oxide", "randomized controlled trial" and "complication"
Cochrane Library	"inhaled nitric oxide", "randomized controlled trial" and "complication"
Wanfang	"吸入一氧化氮" and "随机对照"
